# Efficacy of PD-1 blockade in cervical cancer is related to a CD8^+^FoxP3^+^CD25^+^ T-cell subset with operational effector functions despite high immune checkpoint levels

**DOI:** 10.1186/s40425-019-0526-z

**Published:** 2019-02-12

**Authors:** A. M. Heeren, J. Rotman, A. G. M. Stam, N. Pocorni, A. A. Gassama, S. Samuels, M. C. G. Bleeker, C. H. Mom, H. J. M. A. A. Zijlmans, G. G. Kenter, E. S. Jordanova, T. D. de Gruijl

**Affiliations:** 1 Department of Medical Oncology, Amsterdam UMC, Vrije Universiteit Amsterdam, Cancer Center Amsterdam, De Boelelaan 1117, 1081 HV Amsterdam, The Netherlands; 2 Center for Gynecologic Oncology Amsterdam (CGOA), Amsterdam UMC, Vrije Universiteit Amsterdam, Amsterdam, The Netherlands; 3 Center for Gynecologic Oncology Amsterdam (CGOA), The Netherlands Cancer Institute - Antoni van Leeuwenhoek Hospital, Amsterdam, The Netherlands; 4 Department of Pathology, Amsterdam UMC, Vrije Universiteit Amsterdam, Amsterdam, The Netherlands

**Keywords:** Cervical cancer, T cells, FoxP3, HPV16 E6, PD-1 blockade, Lymph nodes

## Abstract

**Background:**

Cervical cancer (CxCa) is mainly a locally invading disease that metastasizes to loco-regional lymph node basins before involving distant organs in more advanced stages. Local immune potentiation of tumor-draining lymph nodes (TDLN) may thus protect against tumor progression.

**Methods:**

To identify therapeutic targets for local immune modulation, multi-parameter flow cytometric T-cell profiling of primary cervical tumors (PT) and TDLN (*n* = 37) was performed. The in-vitro effect of PD-1 blockade on T-cell reactivity to HPV16 E6 oncoproteins was determined in cultures of TDLN and PT single cell suspensions (*n* = 19). Also, intracellular cytokine staining (ICS) upon anti-CD3 stimulation was performed in metastatic TDLN (LN+) and PT (*n* = 7), as well as multiplexed immunofluorescence histochemistry staining (*n* = 8).

**Results:**

Our data revealed elevated rates of activated regulatory T cells (aTregs) and of central or effector memory CD8^+^ T cells in metastatic TDLN (LN+) as compared to tumor-free TDLN (LN-), and equally high or even higher rates of these subsets in PT. Both memory subsets co-expressed multiple immune checkpoints. PD-1 blockade significantly enhanced detectable E6-specific T-cell responses in 4/5 HPV16+ LN+ and in 1/5 HPV16+ PT. Whereas aTreg rates were higher in anti-PD-1 non-responders, in responders elevated levels of CD8^+^FoxP3^+^CD25^+^ T cells were observed, which correlated with the efficacy of PD-1 blockade (*P* = 0.018). This subset was characterized by an early effector memory phenotype with particularly high levels of co-expressed PD-1, CTLA-4, TIM-3 and LAG-3 checkpoints, but, rather than exhausted, was shown upon polyclonal activation to produce higher levels of Granzyme-B and effector cytokines as compared to its CD8^+^FoxP3^−^ counterparts.

**Conclusion:**

These observations support local PD-(L)1 blockade to interrupt loco-regional immune suppression in CxCa and control metastatic spread to TDLN. Furthermore, our data identify CD8^+^FoxP3^+^CD25^+^ T cells as therapeutic targets, which may also serve as predictive biomarker for PD-(L)1 checkpoint blockade.

**Electronic supplementary material:**

The online version of this article (10.1186/s40425-019-0526-z) contains supplementary material, which is available to authorized users.

## Background

The development of cervical cancer (CxCa) is associated with suppression of CD4^+^ and CD8^+^ T-cell responses against human papillomavirus (HPV) [1, 2]. Even though screening programs and vaccines protecting against high-risk HPV-types have been implemented, CxCa is still the second most diagnosed cancer among women worldwide [3, 4]. The disease is mainly locally invading, with the involvement of parametrium, vagina, bladder and pelvic lymph nodes, rather than of distant organs [5]. The most important prognostic factor in early stage CxCa is the presence of lymph node metastases, with a 5-year survival rate that decreases from circa 80 to 60% in patients with only one tumor-positive node, and this percentage decreases further with the presence of multiple lymph node metastases [6, 7]. In order to improve the prognosis of CxCa patients at risk for (lymph node) metastasis, novel adjuvant immunotherapeutic strategies should be explored focusing on targeting the tumor-draining lymph nodes (TDLN).

Many clinical trials have shown that releasing immunosuppressive brakes on effector T cells, e.g. by blocking inhibitory receptors such as cytotoxic T-lymphocyte-associated antigen 4 (CTLA-4) and programmed cell death (−ligand)-1 (PD-(L)1), leads to unprecedented complete and long-lasting clinical responses in subgroups of patients with various cancer types [8–10]. In squamous cell CxCa, increased expression of (diffuse) PD-L1 on tumor cells is associated with poorer survival [11, 12]. High rates of PD-L1-positive myeloid cells are associated with high rates of regulatory T cells (Tregs) in metastatic cervical lymph nodes [13]. Several trials investigating immune checkpoint blockade in CxCa have been conducted or are ongoing, but mainly in patients with recurrent or metastatic disease [14]. Anti-CTLA-4 therapy showed minimal clinical efficacy in patients with recurrent or metastatic CxCa; out of 42 patients, no complete responses were observed and only one patient had a partial response [15]. Based on a reported overall response rate of 14.3%, of which 2.6% represented complete responses, pembrolizumab was recently approved by the FDA for PD-1 blockade in recurrent or advanced CxCa [16, 17]. In view of these low response rates on systemic immune checkpoint blockade in the metastatic setting, and since CxCa mainly spreads to adjoining tissues and pelvic lymph nodes before hematogenous spread occurs, we hypothesize that locally applied low-dose immunotherapy in earlier stages of the disease may more effectively achieve tumor control while lowering the risk of immune-related side effects associated with systemic administration of checkpoint inhibitors [18, 19].

No reliable biomarkers predictive of clinical response on PD-1 blockade have been identified yet. Although some studies have shown that tumor PD-L1 expression correlates with the anti-tumor response upon PD-L1 checkpoint blockade in different cancer types [20, 21], other studies showed that PD-L1-negative tumors can respond as well [22, 23]. In order to select the appropriate patients who can clinically benefit from immunotherapy, more research focusing on the mechanisms of PD-1 inhibition is needed. Previously, it was thought that PD-1 inhibition was able to reverse exhaustion of chronically antigen-exposed effector T cells [24], however, current data demonstrate that PD-1 blockade acts on earlier activation stages of T cells [25–27]. Conversely, whereas CTLA-4 blockade was previously thought to affect effector T-cell priming, it is now recognized to also effect Treg depletion, thus possibly synergizing with PD-1 blockade in the effector phase [28].

With this study we aimed to identify immunotherapeutic targets on T cells in primary cervical tumors and their draining lymph nodes for local intervention strategies. Immune profiling of primary tumors (PT) and pelvic tumor-draining lymph nodes (TDLN) revealed predominant and elevated PD-1 expression on effector T-cell subsets in PT and metastatic TDLN. This observation prompted the in-vitro functional validation of anti-PD-1 blockade as local treatment strategy for patients with CxCa. Our data suggest more consistent biological efficacy of PD-1 blockade in TDLN than in PT and implicate a specific CD8^+^ T-cell subset with an apparently exhausted phenotype, but with superior effector functions. These findings support the clinical exploration of local PD-(L)1 blockade in early-stage CxCa.

## Methods

### Patients

Patients (*n* = 28) were enrolled in member institutions of the Center for Gynecologic Oncology Amsterdam (CGOA), i.e., the Antoni van Leeuwenhoek (AVL) Hospital and the Amsterdam UMC. Clinical samples were processed in the Amsterdam UMC-Cancer Center Amsterdam (CCA). The study was performed in accordance with the ethical guidelines of the 1975 Declaration of Helsinki and was approved by the local Institutional Review Boards (no. NL25610.058.08). All included patients gave written informed consent. Patients who participated in this study underwent a radical hysterectomy with lymphadenectomy or a lymph node debulking prior to chemoradiotherapy for CxCa. None of the patients were treated with chemoradiotherapy before enrollment. In Table 1 the clinical and pathological characteristics of the study population per subgroup are described. HPV-typing was performed according to the institutional standard procedures. Data per patient are provided in Additional file 1: Table S1.

**Table 1:** Clinical and pathological characteristics of the study population

Clinical characteristics	LN- (*n* = 14)	LN+ (*n* = 13)	PT (*n* = 10)
Age, mean ± SD	42,7 ± 9,5	47,6 ± 16,4	41,9 ± 12,1
FIGO stage			
IB1	6 (42)	2 (15)	7 (70)
IB2	3 (22)	2 (15)	2 (20)
IIA2	1 (7)	1 (8)	1 (10)
IIB	3 (22)	6 (47)	0
IIIB	1 (7)	2 (15)	0
Histology			
SCC	13 (93)	11 (85)	10 (100)
ASCC	1 (7)	2^a^ (15)	0
Vaginal involvement			
Yes	0	2 (15)	1 (10)
No	11 (79)	10 (77)	9 (90)
Unknown	3 (21)	1 (8)	0
Parametrium invasion			
Yes	6 (43)	10 (77)	0
No	7 (50)	3 (23)	10 (100)
Unknown	1 (7)	0	0
HPV-type			
6	0	1 (8)	0
16	7 (50)	7 (53)	5 (50)
18	1 (7)	2 (15)	0
31	1 (7)	0	0
33	0	1 (8)	1 (10)
59	0	0	1 (10)
Unknown	4 (29)	1 (8)	2 (20)
p16+	1 (7)	–	1 (10)
Negative	0	1 (8)	0

Values in the table expressed as n (%) ^a^ Two tumor-positive lymph nodes were collected from the same patient. *Abbreviations*: *LN-* tumor-negative lymph nodes, *LN+* tumor-positive lymph node, *FIGO* International Federation of Gynecology and Obstetrics, *SCC* squamous cell carcinoma, *ASCC* adenosquamous cell carcinoma, *HPV* human papillomavirus, *PT* primary tumor

#### Collection of material and processing

Leukocytes from tumor-negative lymph nodes (LN-, *n* = 14), metastatic lymph nodes (LN+, *n* = 13) and primary tumors (PT, *n* = 10) were isolated as previously described [13]. In short, LNs were bisected and scraped 15 times with a surgical blade. LN cells from the blade were collected in dissociation medium (RPMI1640 (BE12-702F; Lonza), supplemented with 0.1% DNase I (10,104,159,001; Roche), 0.14% Collagenase A (10,103,586,001; Roche), 5% fetal calf serum (FCS; SV30160.03; Hyclone), and Penicillin Streptomycin Glutamine (PSG; 10,378–016; Gibco)). If the PT was of sufficient size, a tumor biopsy was taken. After collection of the samples, the patient material was processed further for routine diagnostic pathology procedures. The PT biopsy was cut into small fragments with a surgical blade and resuspended in dissociation medium. Next, primary tumor and TDLN samples were incubated on a magnetic stirrer for 45 min at 37 °C. For the primary tumor this was repeated twice. Finally, the primary tumor and LN cells were washed with Iscove’s Modified Dulbecco Medium (IMDM; BE12-722F; Lonza) containing 10% FCS, Gentamicin/Amphotericin B (50–0640; Gibco), and PSG, passed through a 100 μm cell strainer (352,360; BD Falcon), erythrocytes were lysed and cells were counted. Cells were either used directly or stored in liquid nitrogen until further use.

#### Multi-color flow cytometry

For phenotypic T cell analysis and comparison of the microenvironment of PT and TDLN, multi-color flow cytometry was carried out using the LSR Fortessa X-20 (BD Biosciences). 150,000 T-cells per sample were stained using the following surface antibodies: CD3, CD4, CD8, CD25, CD45RA, CD27, CD127, HLA-DR, PD-1, TIM-3 and LAG-3. See Additional file 2: Table S2 for antibody specifications. After surface staining, cells were permeabilized and stained for FoxP3, Ki67, and CTLA-4 using a FoxP3 staining kit (00–8332-56, 00–5223-56, 00–5123-43; eBioscience) according to the manufacturer’s instructions. Data were analyzed using Kaluza version 1.3 (Beckman Coulter). CD8^+^ T cell data were visualized in t-Distributed Stochastic Neighbor Embedding (t-SNE) density plots generated in FCS express 6 (De Novo software).

#### HPV16 E6 specific T-cell stimulation with anti-PD-1 and IFNγ analyses

The detection of antigen-specific IFNγ release by PT- or TDLN-derived T cells, was performed using a pool of overlapping 15-mer synthetic long peptides covering the HPV16 E6 sequence (PepMix™ HPV 16 (protein E6) (PM-HPV16-E6; JPT)) as tumor antigen and a CEFT pool (PM-CEFT-3; JPT) as a positive recall control. PT- or TDLN-derived cells were washed and resuspended at 5 × 10^6^/mL in IMDM medium with human AB serum 5% (2,931,949; CELLect™, MP Biomedicals) and PSG and afterwards equally divided into responder and stimulator cells. The stimulator cells served as antigen presenting cells and were loaded with either the HPV16 E6 long peptide pool (1 μg/mL per peptide) or with the CEFT peptide pool (1 μg/mL per peptide) and 3 μg/mL β2-microglobulin (β2M; 30C-cp1022U; Fitzgerald Industries, International). After 1-h incubation at 37 °C, cells were washed with IMDM without human AB and the stimulator cells resuspended at 1 × 10^6^/mL. Stimulator and responder cells (at least 0.5–1 × 10^6^ cells of each) were co-seeded in a 48-well or 24-well plate. IL-2 (10 U/mL; Novartis) and IL-15 (10 ng/mL; 34–8159; eBioscience) were added to each well [29]. Finally, anti-PD-1 (nivolumab, Bristol-Myers Squibb) 10 μg/mL was added to half of the E6 and CEFT wells and the cells were incubated for 10 days. Every 3–4 days the medium was changed with IMDM medium, and IL-2 and IL-15 replenished.

Cells were harvested at day 10 and seeded in 2 × 4–6 replicate wells at a density of 1 × 10^5^/well (E6) or 0.5 × 10^5^/well (CEFT) in a multiscreen 96-well plate coated with an IFNγ catch antibody (3420-2A; Mabtech). Cells were either re-challenged overnight (o/n) with the peptides to which they were initially stimulated or cultured with a DMSO vehicle control. Next day the cells were removed and the plates rinsed and developed according to manufacturer’s instructions (3420-2A; Mabtech). Spots were counted by an automated ELISPOT-reader (AID Diagnostika). IFNγ ELISPOT responses were considered positive when 1) the mean number of spots in the test condition exceeded the mean number of spots in the control condition by at least two-fold; 2) the absolute increase in spots in the test condition was at least 5; and 3) the mean number of spots exceeded the mean number of spots of LN- plus twice the standard deviation.

#### Polyclonal stimulation and intracellular cytokine staining and detection

Four LN+ suspensions and three PT suspensions were used for anti-CD3 stimulation in the presence or absence of anti-PD-1. At least 1,5 × 10^6^ cells were incubated with OKT3 (1:100; 8DSTQ00; Janssen Cilag) for 1 h at 4 °C. After incubation and washing, cells were resuspended in IMDM medium and transferred to a 24-well plate coated with 1:100 affinity-purified goat-anti-mouse Ig (Z0420; DAKO; Agilent) for 1 h at 4 °C. Subsequently, 10 μg/mL anti-PD-1 was added to one of the wells (nivolumab, Bristol-Myers Squibb) and the cells were cultured o/n at 37 °C. The next day, Golgiplug (1:500; 555,029; BD Biosciences) was added and cells were incubated for another 4–5 h at 37 °C. Subsequently, cells were collected and incubated with 1:1000 fixable viability dye eFluor 780 (65–0865-14; eBioscience) in PBS for 30 min at 4 °C. Next, both LN+ as PT samples were stained after o/n anti-CD3 stimulation as described above with the following membrane antibodies: CD3, CD4, CD8 and PD-1 and the following intracellular antibodies: FoxP3, TNFα, IL-2, IFNγ, and Granzyme B (GrB). See Additional file 2: Table S2 for antibody specifications. Flow cytometric analysis was performed as described above.

#### Multiplexed immunohistochemistry

Multiplexed immunofluorescence staining was performed on LN+ (*n* = 4) and PT (*n* = 4) samples from patients with CxCa to identify the location of FoxP3-expressing CD8^+^ T cells.

Formalin-fixed, paraffin-embedded tissue blocks were sectioned at 4 μm and mounted on StarFrost slides. Slides were deparaffinized in 3x xylene and washed in 1 × 100% and 1 × 96% ethanol. Then, endogenous peroxidase was blocked with 0.3% H_2_O_2_ in methanol for 20 min in the dark. Slides were rehydrated in 70% ethanol and Milli-Q water and an extra fixation step was included for 20 min with 10% neutral buffered formalin (3800604E; Leica Biosystems), followed by 2 × 2 min in Milli-Q water and 2 min in 0.05% Tween20 in 1x Tris-buffered saline (TBST). Antigen retrieval microwave treatment was carried out in 0.05% ProClin300/Tris-EDTA buffer at pH 9.0 in an 800 W standard microwave at 100% power until boiling point, followed by 15 min at 30% power. Slides were cooled down, washed in Milli-Q water and 1x TBST and were blocked with Normal Antibody Diluent (S0809; DAKO) for 10 min at room temperature (RT). After that, slides were incubated with primary antibody diluted in Normal Antibody Diluent o/n at RT (anti-PD-1), for 30 min at RT (anti-CD8, anti-CD3, and anti-GrB), and for 60 min at RT (anti-FoxP3) and 30 rounds per minute (rpm) on a shaker. Primary antibodies were separately added and incubated in the following order: anti-PD-1, anti-CD8, anti-CD3, anti-FoxP3, and anti-GrB. See Additional file 2: Table S2 for antibody specifications. Next, slides were washed 3x in 1x TBST at RT and 30 rpm and subsequently incubated with SuperPicture Polymer Detection Kit - HRP - broad spectrum (87–8963; Life Technologies) for 20 min at RT and 30 rpm. Afterwards, slides were washed 3x in 1x TBST and incubated with Opal fluorochromes diluted in amplification buffer (all provided by the OPAL 7-color fIHC kit; NEL797B001KT, Perkin Elmer) for 10 min at RT and 30 rpm: 1:100 Opal520, 1:250 Opal620, 1:150 Opal690, 1:150 Opal570, and 1:250 Opal540, respectively. Next, slides were washed 3x in 1x TBST and a final microwave treatment with AR6 buffer (provided by the OPAL 7-color fIHC kit) was performed and slides were washed in Milli-Q water and in 1x TSBT. NB: all steps were sequentially repeated for each primary antibody. DAPI working solution (provided by the OPAL 7-color fIHC Kit) was applied for 5 min at RT and the slides were washed again in 1x TBST and Milli-Q water, mounted under coverslips with ProLong Diamond antifade mounting medium (P36970; Life Technologies) and stored at 4 °C until imaging.

#### Imaging and quantification

The multiplex immunofluorescence stainings were visualized with Leica TCS SP8 microscope (Leica), tile-scan (3 × 3, 40x oil objective with 1.3 NA) images were generated and viewed using LAS AF Lite software (Leica). Single-layer Tagged Image File Formats were used for automated quantification analysis in TissueStudio® software version 4.3.0 (Definiens). Software training for tissue- (intratumoral vs. peritumoral) and cell segmentation was done based on DAPI staining. Then, Comma Separated Value (.csv) files were extracted and used for image cytometry in FCS express 6 (De Novo software). Gates were set using the (manually) determined threshold for each marker in the TissueStudio software.

#### Statistical analysis

Normal distribution was tested using either the D’Agostino-Pearson omnibus test (in case of sufficient sample size) or the Shapiro-Wilk test. In case of normally distributed data an unpaired two-sided t test was used to compare T-cell frequencies in LN- vs. LN+ and LN+ vs. PT or, otherwise, the Mann-Whitney-U test was used. A paired t-test (for normally distributed data) or the Wilcoxon test was used to compare frequencies of CD8^+^FoxP3^+^ vs. CD8^+^FoxP3^−^ populations and to compare T cell subset frequencies in intratumoral vs. peritumoral areas. Correlations were determined by the Pearson *r* test. Data were analyzed using Prism 7 Software. *P*-values below 0.05 were considered statistically significant.

## Results

### Immunophenotyping of T-cell subsets in cervical cancer (CxCa) tumor-draining lymph nodes (TDLN) and primary tumors (PT) and expression of immune checkpoints

**Fig. 1:**
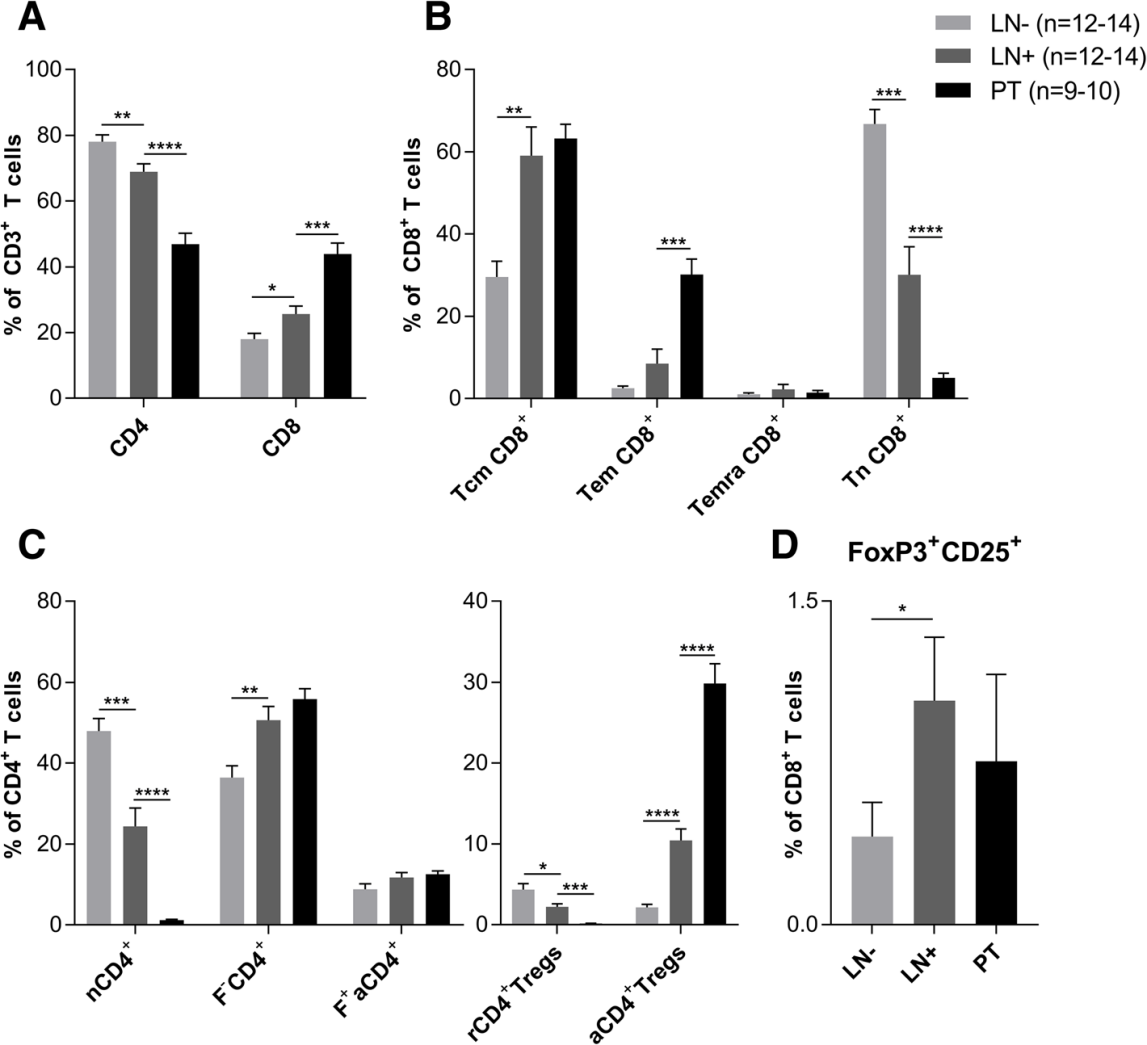
T-cell subset frequencies in LN-, LN+ and PT of patients with CxCa. **a** Frequencies of CD4^+^ and CD8^+^ T cells. **b** Frequencies of CD8^+^ central memory (Tcm, CD27^+^CD45RA^−^), effector memory (Tem, CD27^−^CD45RA^−^), and effector (Temra, CD27^−^CD45RA^+^) T cells. **c** Left panel: frequencies of naïve (nCD4^+^, FoxP3^−^CD45RA^+^), F^−^CD4^+^ (FoxP3^−^CD45RA^−^) and F^+^aCD4^+^ (FoxP3^int^CD45RA^−^) conventional CD4^+^ T cells. Right panel: frequencies of activated (aCD4^+^Tregs, FoxP3^hi^CD45RA^−^) and resting regulatory T cells (rCD4^+^Tregs, FoxP3^int^CD45RA^+^). **d** Frequencies of CD8^+^FoxP3^+^CD25^+^ T cells. Error bars represent standard error of the mean. LN-: *n* = 12–14, LN+: *n* = 12–14, PT: *n* = 9–10. *P = 0.01 to 0.05, **P = 0.001 to 0.01, ***P = 0.001 to 0.0001, ****P < 0.0001

We assessed the frequencies of various T-cell subsets in single-cell suspensions derived from 27 cervical TDLN and 10 PT. As demonstrated in Fig. 1a, a relative shift from CD4^+^ to CD8^+^ T cells was apparent in LN+ as compared to LN-, and significantly more so in PT than in LN+. A decrease in naïve CD8^+^ T cells (Tn) was found in LN+ as compared to LN- (*P* < 0.001; Fig. 1b), and, as expected for an effector site, naïve T-cell rates were even lower in PT (*P* < 0.0001). In PT, an increase of effector memory CD8^+^ T cells (Tem; CD27^−^CD45RA^−^) was found (*P* < 0.001). Increased rates of effector and central memory CD8^+^ T cells (Tcm) in LN+ and PT confirmed our previous data [13], and indicated tumor-associated induction of T-cell differentiation.

For CD4^+^ T-cell populations, frequencies were determined based on CD45RA and FoxP3 expression as previously proposed by Miyara et al. [30], subdividing this group into naïve CD4^+^ T cells (nCD4^+^), memory-like CD4^+^ T cells (F^−^CD4^+^) and cytokine-producing activated CD4^+^ T cells (F^+^aCD4^+^; for gating procedure see Additional file 3: Figure S1A). As expected, predominantly nCD4^+^ (FoxP3^−^CD45RA^+^) were present in LN- (Fig. 1c). Based on CD45RA, FoxP3 and Ki67 expression, activated Tregs (aTregs) were detected at high frequencies in LN+, but even more so in PT (*P* < 0.0001). Resting Tregs (rTregs) were found at the highest frequencies in LN-. These data indicate that rTregs recruited to PT or LN metastases, are rapidly activated in the tumor microenvironment (TME) to become functional aTregs consistent with findings in an earlier report [31]. Although frequencies were low, significantly more CD8^+^FoxP3^+^CD25^+^ T cells were present in LN+ as compared to LN- (*P* = 0.03; Fig. 1d), whereas no significant differences were found in LN+ vs. PT (for gating procedure see Additional file 3: Figure S1B).

**Fig. 2:**
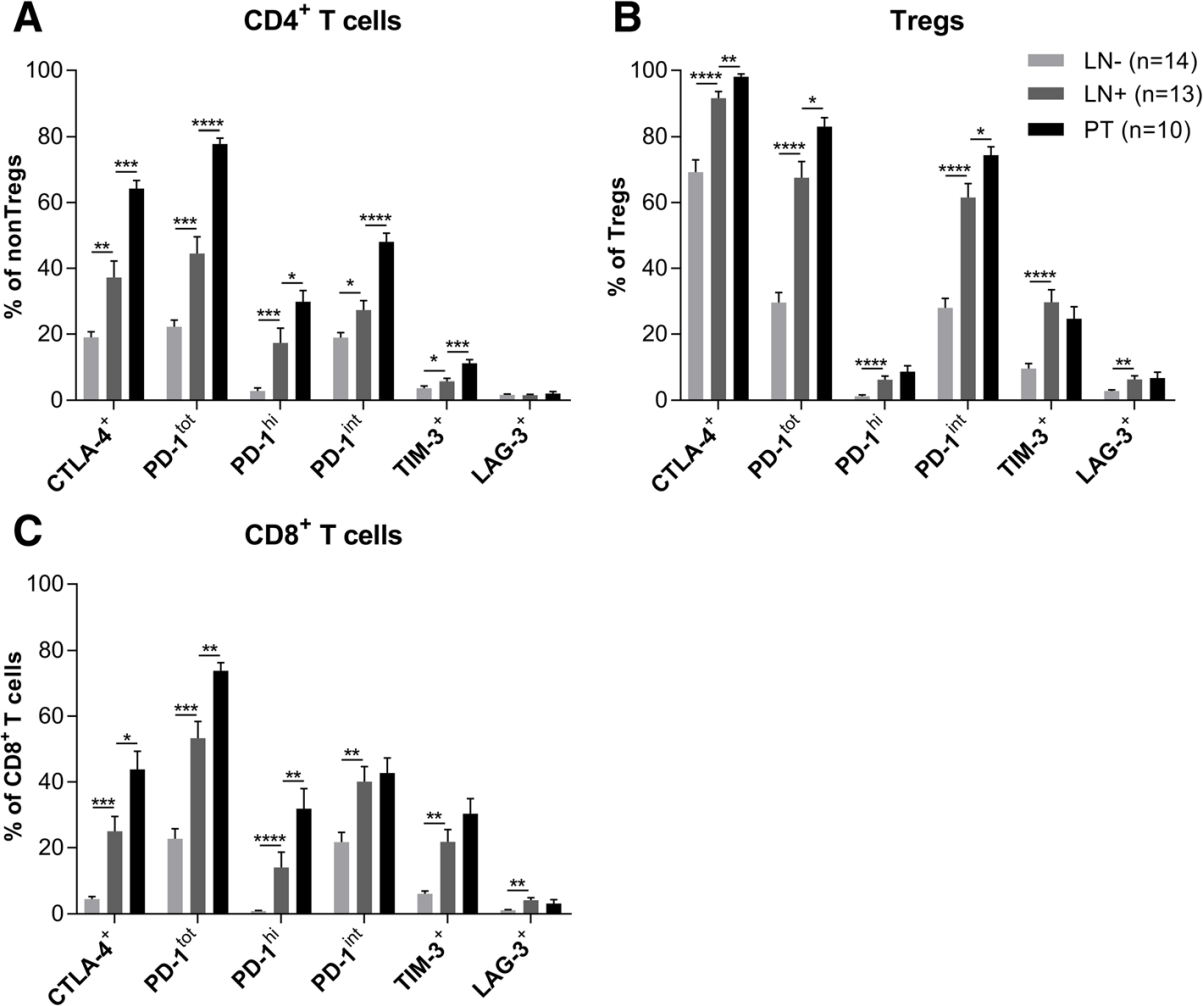
Immune checkpoint expression on CD4^+^ and CD8^+^ T-cell subsets in LN-, LN+ and PT. Expression rates of CTLA-4, total PD-1, intermediate-level and high-level PD-1, TIM-3 and LAG-3 on (**a**) conventional CD4^+^ T cells (non-Tregs) (**b**) regulatory T cells (Tregs), and (**c**) CD8^+^ T cells. Error bars represent standard error of the mean. LN-: *n* = 14, LN+: *n* = 13, PT: *n* = 10. *P = 0.01 to 0.05, **P = 0.001 to 0.01, ***P = 0.001 to 0.0001, ****P < 0.0001

Next, we studied the expression levels of various immune checkpoint receptors on the different T-cell subsets (i.e., CD4^+^ and CD8^+^ T cells and Tregs). See Additional file 4: Figure S2 A-B for gating strategy of immune checkpoints on CD4^+^ and CD8^+^ T cells. For all studied immune checkpoints (i.e., CTLA-4, PD-1, TIM-3, and LAG-3) on all three assessed T-cell subsets, the expression levels were significantly higher in LN+ vs. LN-, except for LAG-3 on CD4^+^ T cells. Generally, immune checkpoint expression levels on these T-cell subsets were even higher in PT than in LN+ (Fig. 2a-c). As expected, the highest expressed immune checkpoint on Tregs was CTLA-4 (Fig. 2b), whereas on conventional CD4^+^ T cells the highest averaged expression rate was found for PD-1 (Fig. 2a). Also on CD8^+^ T cells PD-1 was the most frequently expressed immune checkpoint (Fig. 2c). PD-1 expression levels on Tregs were mainly intermediate, whereas in the conventional effector subsets relatively more cells had high PD-1 expression levels (Fig. 2a-c). Nevertheless, CD8^+^ T cells with intermediate PD-1 levels outnumbered CD8^+^ T cells with high expression levels in LN+; a more equal distribution was observed in PT (Fig. 2c). Of note, on CD8^+^PD-1^hi^ T cells also higher expression levels of the other immune checkpoints were observed (Additional file 4: Figure S2C) as compared to the CD8^+^PD-1^int^ T cells (Additional file 4: Figure S2D). Moreover, checkpoints were often co-expressed on conventional CD8^+^ T cells in both CxCa PT and LN+ (Additional file 4: Figure S2E). Whereas combined expression of PD-1 and other checkpoints (TIM-3 and LAG-3 in particular) has been identified as a sign of functional exhaustion [24], intermediate expression levels of PD-1 has been related to recoverable effector functionality through PD-(L)1 blockade [25–27]. Considerable frequencies of CD8^+^PD-1^int^ T cells in PT and LN+ therefore provided us with a rationale to explore whether PD-1 blockade could enhance antitumor T-cell functions.

### HPV-specific T-cell cultures with PD-1 blockade

**Fig. 3:**
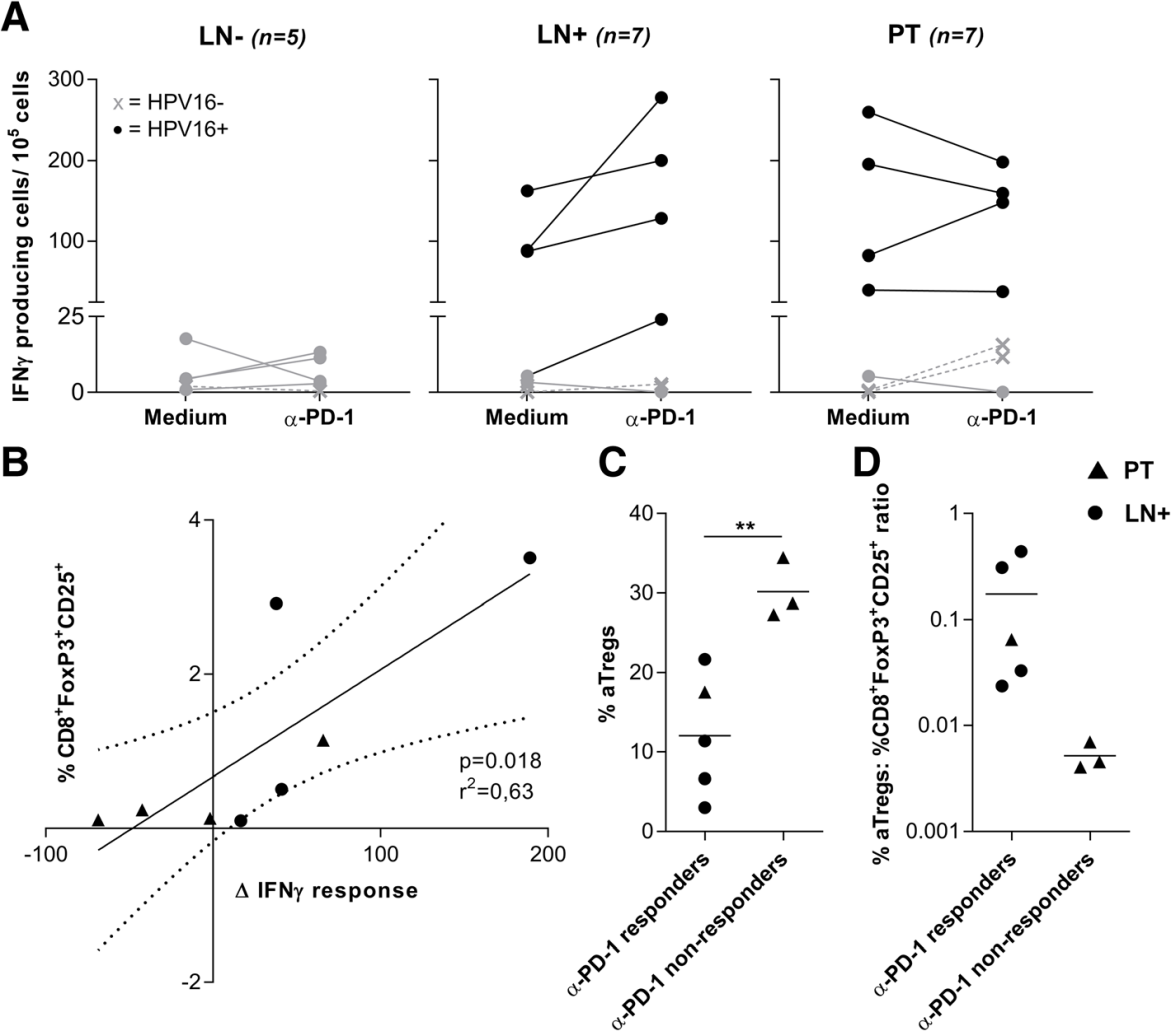
In-vitro PD-1 blockade in HPV16 E6-stimulated LN-, LN+ and PT single cell suspensions. The IFNγ ELISPOT reactivity is expressed as number of spots per 100,000 T cells. **a** IFNγ T cell reactivity to a set of 15-mer overlapping peptides covering the HPV16 E6 protein sequence; enhanced reactivity upon PD-1 blockade was observed in 4/4 responders in the LN+ (*n* = 7) group and in 1/4 responders in the PT (*n* = 7) group; there were no positive responders in the LN- (*n* = 5) group. Data from positive responders are depicted in black, from non-responders in grey. HPV-types other than 16 are depicted in the graph (HPV16-, **x**; HPV16+, ●) (**b**) The IFNγ Elispot response upon PD-1 blockade correlated with the percentage of CD8^+^FoxP3^+^CD25^+^ T cells in the tested samples. Only positive responders to HPV16 E6 were included in the analysis. **c** Activated Treg frequencies (aTregs) and (**d**) the ratio of CD8^+^FoxP3^+^CD25^+^ T cells: Tregs, both subdivided by responders to anti-PD-1 (i.e., increased HPV16 E6-specific IFNγ response upon PD-1 blockade) and non-responders to anti-PD-1 in HPV16+ LN+ and PT samples (with detectable reactivity to HPV16 E6, same samples as shown in panel **b** and **c**). Error bars represent standard error of the mean. **P = 0.001 to 0.01

To assess PD-1 blockade efficacy, we performed in-vitro tumor-antigen specific T-cell cultures with overlapping 15-mer HPV16 E6 peptides in the absence or presence of nivolumab (10 μg/mL). We initially performed these cultures o/n (*n* = 9), followed by IFNγ ELISPOT, but we were unable to detect any robust E6-specific T-cell responses in the tested single-cell suspension samples, unlike CEFT recall responses which were readily detected (data not shown). We therefore resorted to a 10-day culture method, followed by an o/n IFNγ ELISPOT assay (as previously described by Yuan and colleagues [29]). Employing this method, we were able to detect HPV16 E6 specific responses in 3/5 LN+ samples derived from patients with HPV16+ CxCa, and in 4/5 HPV16+ PT samples, but in none of the four tested HPV16+ LN- samples (Fig. 3a). The specificity of our assay was confirmed by the fact that we did not measure any HPV-16 E6-specific T-cell responses in any of the other samples that were derived from patients with HPV infections other than HPV16 (Additional file 5: Figure S3A). Significantly increased E6-specific T-cell frequencies after PD-1 blockade in positive E6 responders were observed in 4/4 LN+, but, remarkably, in only 1/4 PT samples (Fig. 3a). These effects were restricted to tumor antigen-specific T-cells as CEFT recall responses were also detected in HPV16-negative samples and not increased by PD-1 blockade (Additional file 5: Figure S3B).

### CD8^+^FoxP3^+^CD25^+^ T cell and aTreg rates in relation to PD-1 blockade efficacy

Multiparameter flow cytometric analysis of the HPV16+ samples with positive E6 responses before stimulation with HPV16 E6 peptides(LN+ *n* = 4, PT *n* = 4), revealed that only one T-cell subset significantly correlated with in-vitro efficacy of PD-1 blockade, i.e., CD8^+^FoxP3^+^CD25^+^ T cells (*P* = 0.018; Fig. 3b). In contrast, aTreg rates were elevated at the start in samples that did not respond to PD-1 blockade (*P* < 0.01, Fig. 3c). Consequently, CD8^+^FoxP3^+^CD25^+^ T cell:aTreg ratios appeared to have predictive value for PD-1 efficacy, although not significant due to small sample size, in LN+ and PT samples with detectable (i.e., pre-existent) E6-specific T-cell responses (Fig. 3d).

### Phenotypic and functional profiling of CD8^+^FoxP3^+^CD25^+^ T cells

**Fig. 4:**
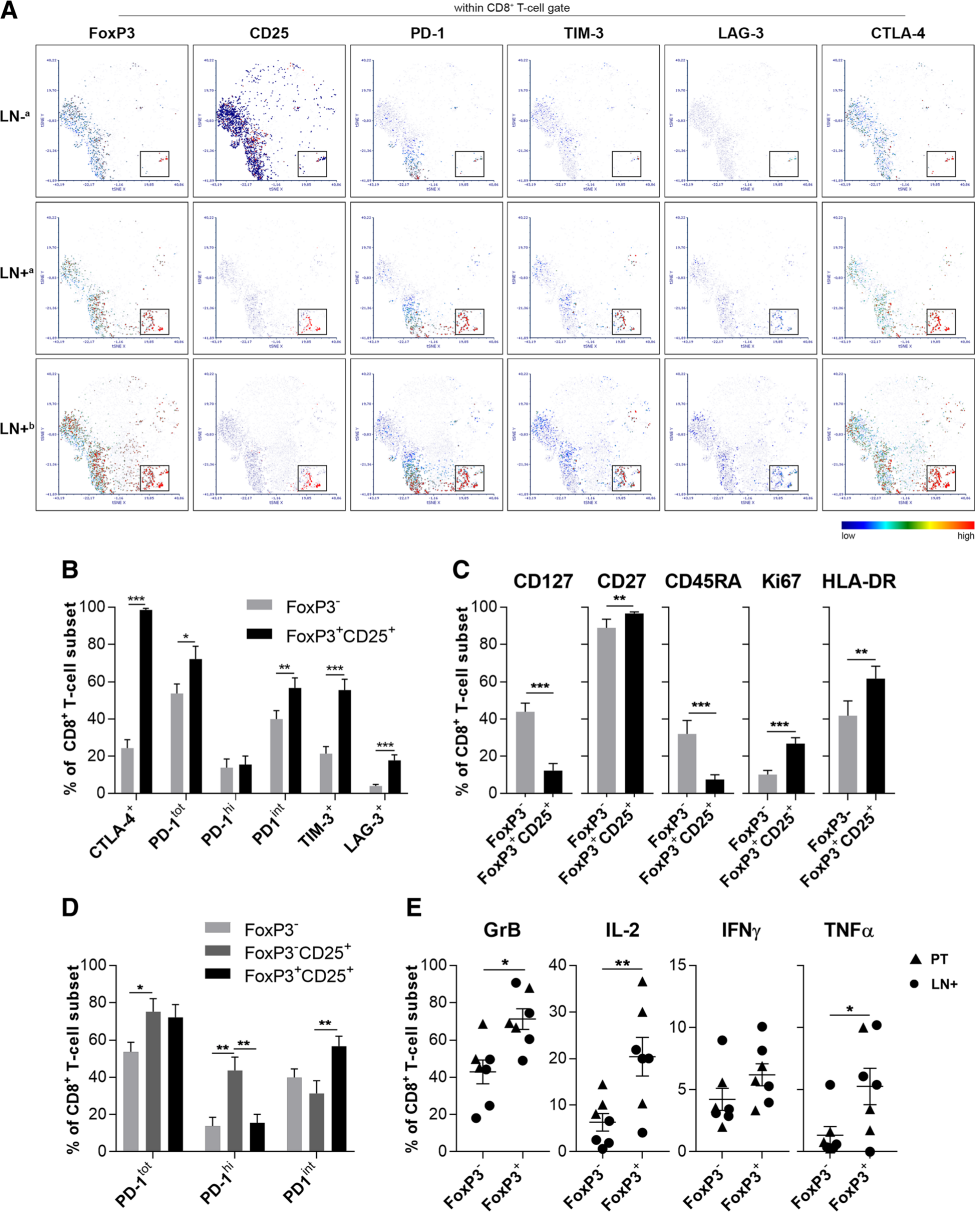
Phenotyping and functional status of CD8^+^FoxP3^+^CD25^+^ T cells vs. CD8^+^FoxP3^−^ T cells. **a** t-SNE density plots showing expression distribution within the CD8^+^ T cell population of FoxP3, CD25, PD-1, TIM-3, LAG-3, and CTLA-4; representative results from matched LN- and LN+ samples from the same patient (^a^) are shown and from another SLN+ sample (^b^). NB: both LN+ samples showed positive response to HPV16 E6, which was enhanced by PD-1 blockade (no response in LN-). Box indicates the CD8^+^FoxP3^+^CD25^+^ population. **b** Immune checkpoint receptor profiling of CD8^+^FoxP3^+^CD25^+^ T cells versus CD8^+^FoxP3^−^ T cells (LN+, *n* = 13). **c** Frequency of the effector and memory markers CD127, CD27 and CD45RA on the CD8^+^FoxP3^−^ vs. CD8^+^FoxP3^+^CD25^+^ subsets in LN+ (*n* = 12). **d** PD-1 expression on CD8^+^FoxP3^−^, CD8^+^FoxP3^−^CD25^+^, and CD8^+^FoxP3^+^CD25^+^ T cells in LN+ (*n* = 13). **e** Frequency of CD8^+^FoxP3^+^ vs. CD8^+^FoxP3^−^ T cells (co-)expressing intracellular Granzyme-B (GrB), IFNγ, IL-2 and TNFα upon o/n anti-CD3 stimulation in LN+ (*n* = 4) and PT (*n* = 3). Error bars represent standard error of the mean. *P = 0.01 to 0.05, **P = 0.001 to 0.01, ***P = 0.001 to 0.0001

To visualize (co-)expression of FoxP3, CD25, PD-1, TIM-3, LAG-3, and CTLA-4 at the single-cell level within the CD8^+^ T cell population of LN+ vs. LN-, high-dimensional t-SNE analysis was performed on flow cytometric data from two pairs of matched LN- and LN+, of which the LN+ had shown elevated HPV-16 E6-specific IFNγ responses after PD-1 blockade. Differential expression of these markers between LN- and LN+ was clear, with a distinct CD25^+^FoxP3^+^ population, co-expressing relatively high levels of the assessed immune checkpoints, present only in the LN+ samples (Fig. 4a). Conventional FACS analyses further confirmed co-expression of multiple immune checkpoints on CD8^+^FoxP3^+^CD25^+^ T cells (Additional file 4: Figure S2F) and, in a comparative analysis with conventional CD8^+^FoxP3^−^ T cells, showed the levels of CTLA-4, TIM-3 and LAG-3 in LN+ to be significantly higher (all *P <* 0.01), but, remarkably, only rates of cells with intermediate PD-1 expression levels were similarly elevated in the CD8^+^ CD25^+^FoxP3^+^ T-cell population (*P* < 0.01, Fig. 4b). CD8^+^FoxP3^+^CD25^+^ T cells in LN+ (and in PT, data not shown) displayed a CD127^−^, CD45RA^−^ and CD27^+^ profile, consistent with a previously reported early effector memory phenotype [32], and were shown to have proliferative capacity and to be activated, as evidenced by increased Ki67 and HLA-DR expression levels, respectively (Fig. 4c). CD25 levels within the CD8^+^ T-cell subset appeared to be generally lower in the LN- than in the LN+ samples (Fig. 4a); within the activated CD8^+^CD25^+^ T cells in LN+ samples, FoxP3^+^ T cells comprised significantly lower frequencies of PD-1^hi^ cells and significantly higher frequencies of PD-1^int^ cells than their FoxP3^−^ counterparts (Fig. 4d).

As CD8^+^FoxP3^+^CD25^+^ T cells in CxCa metastatic tumors were previously reported to be immune suppressive [33] and as expression of multiple immune checkpoints is generally regarded as a sign of exhaustion [24], we assessed the functional status of this subset after polyclonal stimulation. After o/n anti-CD3 stimulation of single-cell suspensions from LN+ (*n* = 4) and PT samples (*n* = 3), intracellular flow cytometric analysis showed increased expression of GrB (*P <* 0.05), IL-2 (*P* < 0.01), and TNFα (*P* < 0.05) in CD8^+^FoxP3^+^ T cells as compared to the conventional CD8^+^FoxP3^−^ T cells (Fig. 4e, Additional file 6: Figure S4A-B). Of note, no such effector activity was observed for CD4^+^FoxP3^+^ Tregs (Additional file 6: Figure S4C-F). Although saturating binding of PD-1 was demonstrated by flow cytometry in parallel cultures with anti-PD-1, no resulting functional changes were observed in this model system (data not shown).

### Phenotype, distribution, and localization of CD8^+^FoxP3^+^ T cells in cervical cancer (CxCa) tissue

**Fig. 5:**
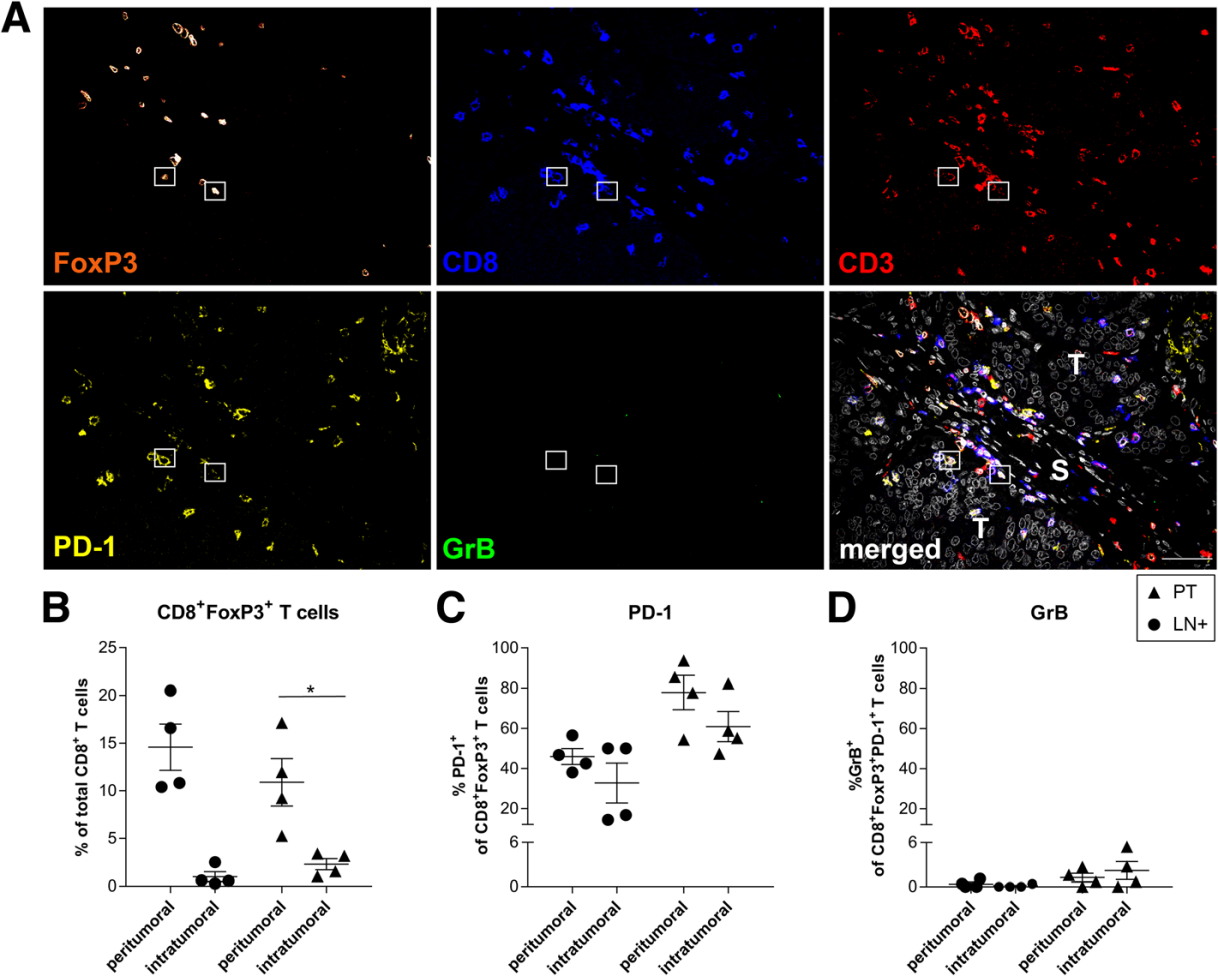
Phenotype, distribution, and localization of CD8^+^FoxP3^+^ T cells in cervical tumor tissue. **a** Representative immunofluorescence staining showing FoxP3 (in orange), CD8 (in blue), CD3 (in red), PD-1 (in yellow), GrB (in green), and the merged image including DAPI (in grey). Boxes denote CD8^+^FoxP3^+^ T cells. Scale bar in merged image is 50 μm. In merged image, T (tumor) represents intratumoral area and S (stroma) represents peritumoral area. Scatter plot showing peritumoral and intratumoral rates for **b** CD8^+^FoxP3^+^ T cells, and percentages of **c** PD-1-expressing CD8^+^FoxP3^+^ T cells and **d** GrB-expressing CD8^+^FoxP3^+^PD-1^+^ T cells in tumor-positive lymph nodes (*n* = 4 for LN+, ●) and primary tumors (*n* = 4 for PT, ▲). Error bars represent standard error of the mean. *P = 0.01 to 0.05

Next, we studied the distribution and localization of CD8^+^FoxP3^+^ T cells in cervical LN+ (*n* = 4) and PT (*n* = 4) samples using the markers CD3, CD8, FoxP3, PD-1, and GrB in a multiplexed immunohistochemistry analysis (Fig. 5a). CD8^+^FoxP3^+^ T cells were mainly located in the peritumoral compartment of PT (*P* = 0.043) and LN metastases (Fig. 5b). Circa 40–60% of CD8^+^FoxP3^+^ cells were positive for PD-1 (PT > LN+), with comparable expression levels in peritumoral and intratumoral T cells (Fig. 5c). Further sub-phenotyping showed low percentages of GrB-expressing CD8^+^FoxP3^+^PD-1^+^ T cells, indicative of a functionally suppressed state of this subset in vivo (Fig. 5d).

## Discussion

The goal of the current study was to profile T-cell populations in CxCa PT and TDLN and identify therapeutic targets for local immune intervention strategies in a bid to curtail tumor progression and metastatic spread. Based on our previous data on PD-L1 expression in cervical PT and TDLN samples [12, 13, 34] and our current data on high PD-1 expression on different T-cell subsets in TDLN and PT, the effects of in-vitro blocking of PD-1 were studied and linked to a CD8^+^ T-cell subset with superior effector functionality.

Cervical tumor development was associated with T-cell differentiation and activation, as evidenced by elevated frequencies of memory T cells, co-expressing multiple immune checkpoints, in LN+ and PT samples. Indeed, we observed high expression of immune checkpoint molecules on the different T-cell subsets in cervical PT and metastatic TDLN. CTLA-4 and PD-1 were most often (co-)expressed, TIM-3 was expressed frequently whereas LAG-3 had low expression rates on most of the T-cell subsets. Checkpoint expression levels were elevated in LN+ over LN- samples and highest in PT samples, indicative of a tissue-specific hierarchy. This is in agreement with findings described previously by Ahmadzadeh et al. in melanoma [35]. Taken together, these results suggest that the TME plays an important role in the differentiation of T cells and upregulation of the checkpoint receptors. Of interest in this regard are the observed increased frequencies of both aTregs and CD8^+^ effector T cells in PT and LN+ with intermediate expression levels of PD-1, which may be particularly amenable to PD-1 blockade [27]. Thus, expansion and/or activation of recruited Tregs and effector cytotoxic T cells to the TME in PT and TDLN may be modulated by PD-(L)1 blockade. Indeed, the inability to affect T cell responses upon HPV16 E6-specific or polyclonal stimulation in o/n cultures of PT and TDLN samples, but the finding of enhanced HPV16 E6 responses after 10 days of culture in the presence of anti-PD-1, suggest a requirement for more long-term conditioning of the microenvironment and a possible expansion or contraction of targeted immune subsets.

We found an association between the IFNγ T-cell response upon stimulation with tumor-specific HPV16 E6 peptides and PD-1 blockade, and lower levels of aTregs as well as elevated levels of a particular subset of CD8^+^ T cells, specifically CD8^+^FoxP3^+^CD25^+^ T cells. Although frequencies were generally low, higher percentages of this CD8^+^ T-cell subset were present in the (metastatic) tumor samples than in LN-, confirming our previously published data [13]. With low expression of CD127 and CD45RA, and high expression of CD27, this subset appeared to have an early effector memory phenotype, more often positive for proliferation (Ki67) and activation (HLA-DR) markers than its CD8^+^FoxP3^−^ counterparts. Interestingly, the subset also had high surface expression levels of immune checkpoints (PD-1, CTLA-4, TIM-3, LAG-3), suggestive of functional exhaustion [36]. In keeping with this notion it did not express GrB in situ. However, these seemingly exhausted cells were actually able to respond to in-vitro CD3-mediated stimulation by expressing GrB and TNFα, and seemed able to be reinvigorated by PD-1 blockade in a 10-day culture experiment. Interestingly, CD8^+^FoxP3^+^CD25^+^ T cells had more often intermediate-level PD-1 expression compared with CD8^+^FoxP3^−^ T cells, suggesting that functionality of these cells is “recoverable” and can be reinvigorated upon anti-PD-1 therapy [37]. Of note, in contrast with the CD8^+^FoxP3^+^CD25^+^ population, CD8^+^FoxP3^−^CD25^+^ T cells did not show a significant correlation with the IFNγ response boosted upon antigen specific stimulation in the presence of anti-PD-1. An explanation for this could be the significantly lower intermediate-level PD-1 expression by CD8^+^FoxP3^−^CD25^+^ T cells and elevated high-level PD-1 expression (Fig. 4d), which may be a sign of true, irreversible exhaustion.

CD8^+^FoxP3^+^ T cells were previously described as CD8^+^ ‘Tregs’ with suppressive functions in CxCa [33] and in other tumor types [38–40]. However, no unique surface marker has been established yet to isolate the population and confirm its suppressive features. There is contrasting evidence suggesting that this subset is a tumor-reactive effector cell population [32, 41, 42]. In melanoma [41] and in non-small-cell lung cancer (NSCLC) [32], CD8^+^FoxP3^+^ T cells were described as a population of early effector T cells. Tassi et al. [32] described that the CD8^+^FoxP3^+^ T cells were PD-1-positive and had an activated non-exhausted phenotype, with a high expression of other immune checkpoint molecules. Of note, the CD8^+^FoxP3^+^ T-cell subset was the only subset able to produce IFNγ after co-culture with autologous lung cancer cells. The authors suggested that these cells may represent a valuable biomarker for monitoring ongoing adaptive immunity in the tumor. Our finding of this subset being present in LN+ with detectable HPV16 E6 reactive T cells, but not in LN-, in which we failed to detect any such responses, is consistent with this suggestion. In addition, Anichini et al. described that these proliferating “early effector cells” could be tumor-reactive effector cells in melanoma in the earliest stage after priming. On the other hand, as upregulation of immune checkpoints is described in T cells chronically stimulated by antigens, the high expression of the immune checkpoints on CD8^+^FoxP3^+^ T cells might also indicate that this is a more differentiated antigen-experienced population, which was also suggested by Le et al. [42]. In the latter study, CD8^+^ T cells became FoxP3^+^ after they had been transferred into tumor-bearing mice, signifying that these cells could serve as a marker of an effective T-cell response in the TME. Moreover, CD8^+^FoxP3^+^ T cells were induced in vitro in co-cultures with breast cancer and ovarian cancer cell lines, whereas in the presence of non-cancerous cells this subset was not induced [43, 44]. These in-vitro differentiated FoxP3-expressing CD8^+^ T cells produced more IFNγ and TGF-β1, whereas no IL-10 secretion was observed [44]. Based on these studies and our own data we propose that these CD8^+^FoxP3^+^ T cells likely represent tumor-specific effector T cells, recruited to the tumor sites, rather than suppressive Tregs. Our immunohistochemical T-cell data shows these effector cells to be mostly situated in the peritumoral areas of both cervical PT and metastatic lymph node tissues. Therefore, the question remains if these cells first need to be recruited into the actual tumor areas before they can exert antitumor immunity after checkpoint inhibition. High frequencies of aTregs may also interfere with their functionality and account for their low in-situ GrB levels, as established by immunohistochemistry. This is in keeping with our observation of an association of the CD8^+^FoxP3^+^CD25^+^ T cell:aTreg ratio with the in-vitro efficacy of PD-1 blockade. As both subsets co-express high rates of CTLA-4 and intermediate-level PD-1, this again argues in favor of a combined anti-PD-1/anti-CTLA-4 intervention strategy.

The effect of PD-1 blockade was more pronounced in LN+ than in PT. This is an interesting observation for which at least two possible explanations exist. First, as described in this study, we observe a tissue-specific hierarchy for expression levels of the different immune checkpoints on T-cells, which is consistent with the notion of a more profound state of T-cell immune suppression or “exhaustion” in PT. Moreover, the favorable PD-1^int^ to PD-1^hi^ ratio on CD8^+^ T cells in LN+ as compared to PT (Fig. 2c), and on CD8^+^FoxP3^+^CD25^+^ T cells in particular (Fig. 4d), is also consistent with a more “recoverable” state of exhaustion, e.g. by PD-1 blockade, as suggested by several recent reports [25, 27, 45]. Secondly, aTregs, i.e., functionally suppressive Tregs [30], are dramatically elevated in PT over LN+, and elevated aTreg rates were associated with the absence of a response to PD-1 blockade (Fig. 3d). Based on this evidence, we conclude that whereas PD-1 blockade may be sufficient to boost antitumor immunity in TDLN, in PT this may additionally require depletion of aTregs, e.g. by administration of anti-CTLA-4. The latter is further supported by the high co-expression levels of PD-1 and CTLA-4 on CD8^+^FoxP3^+^CD25^+^ T cells (Additional file 4: Figure S2F). Our finding of anti-PD-1 blockade efficacy in TDLN also fits with a recent report from Chamoto and colleagues who showed in a mouse model that antitumor efficacy of PD-1 blockade depended on continuous recruitment of effector-memory CD8^+^ T cells, which were induced to proliferate by PD-1 inhibition [46], from TDLN to tumor sites. The CD8^+^FoxP3^+^CD25^+^ early effector-memory T-cell subset in LN+, identified by us as related to PD-1 blockade efficacy, displayed an activated and proliferative state and might well represent the human equivalent of the TDLN-derived effector-memory CD8+ T cells shown to be crucial for effective in-vivo PD-1 blockade.

## Conclusion

In conclusion, our data support the earlier reports of an HPV-specific T-cell repertoire in TDLN “poised for action” and show it to be a valid target for PD-1 blockade [47]. These T cells may subsequently effect both loco-regional and systemic tumor control, thus prolonging (recurrence-free) survival. Moreover, they point to CD8^+^FoxP3^+^CD25^+^ T cells in TDLN as likely therapeutic target, which (together with aTregs) may ultimately also serve as predictive biomarker. These observations provide important supportive evidence for the use of local PD-(L)1 blockade in lifting loco-regional immune suppression in CxCa and controlling metastatic spread to TDLN, one of the most important risk factors in CxCa. Indeed, based on our previous reports [13, 48] and further supported by our current findings, we are testing a neo-adjuvant immunotherapy approach in early-stage CxCa patients, involving intratumoral delivery of anti-PD-L1/durvalumab (who.int/trialsearch, NTR6119) [49]. Importantly, immune monitoring of this trial may provide in-vivo validation of the in-vitro findings from this paper.

## Additional files

Additional file 1: **Table S1.** Clinical characteristics of the study population, per patient. (DOCX 26 kb)

Additional file 2: **Table S2.** Antibody specifications. (DOCX 13 kb)

Additional file 3: **Figure S1.** Gating strategies. (PDF 159 kb)

Additional file 4: **Figure S2.** Co-expression of immune checkpoint receptors on CD8^+^ T-cell subsets. (PDF 1990 kb)

Additional file 5: **Figure S3.** The IFNγ ELISPOT reactivity in LN-, LN+ and PT single cell suspensions. (PDF 380 kb)

Additional file 6: **Figure S4.** Cytokine expression upon anti-CD3 stimulation: CD8^+^ vs. CD4^+^ T cells. (PDF 822 kb)

## Abbreviations

(a)Tregs: (Activated) regulatory T cells
AVL: Antoni van Leeuwenhoek
CCA: Cancer Center Amsterdam
CGOA: Center for Gynecologic Oncology Amsterdam
CTLA-4: Cytotoxic T-lymphocyte-associated antigen 4
CxCa: Cervical cancer
F^+^aCD4^+^: Cytokine-producing activated CD4^+^ T cells
F^-^CD4^+^: Memory-like CD4^+^ T cells
FCS: Fetal calf serum
FMO: Fluorescence minus one
GrB: Granzyme B
ICS: Intracellular cytokine staining
IMDM: Iscove’s Modified Dulbecco Medium
LN-: Tumor-free TDLN
LN+: Metastatic TDLN
nCD4^+^: Naïve CD4^+^ T cells
NSCLC: Non-small-cell lung cancer
o/n: Overnight
PD-(L)1: Programmed cell death (-ligand)-1
PSG: Penicillin streptomycin glutamine
PT: Primary tumor
Rpm: Rounds per minute
RT: Room temperature
rTregs: Resting Tregs
TBST: Tris-buffered saline
Tcm: Central memory CD8^+^ T cells
TDLN: Tumor-draining lymph node(s)
Tem: Effector memory CD8^+^ T cells
TME: Tumor microenvironment
Tn: Naïve CD8^+^ T cells
t-SNE: t-Distributed Stochastic Neighbor Embedding
β2M: β2-microglobulin
