# Supplementary material for: Efficacy of PD-1 blockade in cervical cancer is related to a CD8+FoxP3+CD25+ T-cell subset with operational effector functions despite high immune checkpoint levels
Source: J Immunother Cancer. 2019 Feb 12;7:43. doi: 10.1186/s40425-019-0526-z (PMC6373123; doi:10.1186/s40425-019-0526-z)
Supplement: Supplementary file 1 — Table S1. Clinical characteristics of the study population, per patient. (DOCX 26 kb) [file 40425_2019_526_MOESM1_ESM.docx]

| Supplementary Table 1  Clinical characteristics of the study population, per patient | | | | | | | | | | Collected tissue | | | |
| --- | --- | --- | --- | --- | --- | --- | --- | --- | --- | --- | --- | --- | --- |
| No. | **Age** | **FIGO  stage** | **Histo-logy** | **PT Size (mm)** | **VI**^†^ | **PI**^†^ | **Presence of LN+ (*n*)** | | **HPV-type** | **LN- (*n*)** | **LN+ (*n*)** | **Mets. Size (mm)** | **PT** |
| 1. | 49 | IB1 | SCC | 15 | No | No | | - | N/A | 1 | - |  | - |
| 2. | 36 | IB1 | SCC | 14 | No | No | | - | N/A | 1 | - |  | Yes |
| 3. | 44 | IIIB | SCC | 80 | No | Yes | | - | 16 | 1 | - |  | - |
| 4. | 29 | IIB | ASCC | 40 | No | Yes | | 11 | 18 | 1 | 2 | 50/55 | - |
| 5. | 41 | IB2 | SCC | 38 | No | No | | - | 16 | 1 |  |  | - |
| 6. | 59 | IIB | SCC | 38 | No | Yes | | 1 | 16 | 1 | 1 | 34 | - |
| 7. | 28 | IB1 | SCC | 42 | No | No | | - | p16+ | 1 | - |  | Yes |
| 8. | 50 | IB2 | SCC | 50 | No | Yes | | - | 16 | 1 | - |  | - |
| 9. | 41 | IB1 | SCC | 35 | No | N/A | | - | 31 | 1 | - |  | - |
| 10. | 37 | IB2 | SCC | 50 | No | No | | 2 | 16 | 1 | 1 | 50 | - |
| 11. | 50 | IB1 | SCC | 5 | No | No | | - | N/A | 1 | - |  | - |
| 12. | 42 | IB1 | SCC | 30 | No | Yes | | - | 16 | 1 | - |  | - |
| 13. | 59 | IIB | SCC | 37 | No | Yes | | 2 | 16 | 1 | 1 | 10 | - |
| 14. | 33 | IIA2 | SCC | 32 | No | No | | 5 | N/A | 1 | - |  | Yes |
| 15. | 68 | IIIB | SCC | 70 | No | Yes | | 10 | 33 | - | 1 | 25 | - |
| 16. | 28 | IIA2 | SCC | 60 | Yes | Yes | | 3 | 16 | - | 1 | 38 | - |
| 17. | 41 | IB2 | SCC | 45 | No | Yes | | 2 | Neg | - | 1 | 25 | - |
| 18. | 67 | IIB | SCC | 60 | Yes | Yes | | 4 | N/A | - | 1 | 40 | - |
| 19. | 60 | IIB | SCC | 25 | No | Yes | | 3 | 16 | - | 1 | 40 | - |
| 20. | 69 | IB1 | SCC | 30 | No | No | | 2 | 6 | - | 1 | 1,3 | - |
| 21. | 43 | IB1 | SCC | 33 | No | No | | 1 | 16 | - | 1 | 0,35 | Yes |
| 22. | 30 | IIIB | SCC | 60 | No | Yes | | 3 | 16 | - | 1 | N/A | - |
| 23. | 37 | IB2 | SCC | 62 | Yes | No | | - | 16 | - | - |  | Yes |
| 24. | 42 | IB2 | SCC | 48 | No | No | | - | 59 | - | - |  | Yes |
| 25. | 36 | IB1 | SCC | 30 | No | No | | - | 16 | - | - |  | Yes |
| 26. | 49 | IB1 | SCC | 20 | No | No | | - | 16 | - | - |  | Yes |
| 27. | 43 | IB1 | SCC | 35 | No | No | | - | 16 | - | - |  | Yes |
| 28. | 72 | IB1 | SCC | 24 | No | No | | - | 33 |  |  |  | Yes |

Abbreviations: FIGO, International Federation of Gynecology and Obstetrics; SCC, squamous cell carcinoma; ASCC, adenosquamous cell carcinoma; VI, vaginal involvement; PI, parametrial invasion; HPV, human papillomavirus; N/A, not available; LN-, tumor-negative lymph node; LN+, tumor-positive lymph node; Mets., metastasis; PT, primary tumor. ^†^ When data on VI and PI was not available from the pathology report (e.g. in case of lymph node debulking only), this was obtained from the medical record.
