# Supplementary material for: Efficacy of PD-1 blockade in cervical cancer is related to a CD8+FoxP3+CD25+ T-cell subset with operational effector functions despite high immune checkpoint levels
Source: J Immunother Cancer. 2019 Feb 12;7:43. doi: 10.1186/s40425-019-0526-z (PMC6373123; doi:10.1186/s40425-019-0526-z)
Supplement: Supplementary file 2 — Table S2. Antibody specifications. (DOCX 13 kb) [file 40425_2019_526_MOESM2_ESM.docx]

| **Antibodies** | **Clone** | **Isotype** | **Catalog no.** | **Manufacturer** |
| --- | --- | --- | --- | --- |
| CD3 PerCP-Cy5.5 | SK7 | mouse IgG1 | 332771 | BD |
| CD4 AF700 | RPA-T4 | mouse IgG1 | 557922 | BD |
| CD8 V500 | SK1 | mouse IgG1 | 561618 | BD |
| CD25 APC | 2A3 | mouse IgG1 | 340907 | BD |
| CD45RA APC-H7 | HI100 | mouse IgG2b | 560674 | BD |
| CD27 PE-Cy7 | M-T271 | mouse IgG1 | 560609 | BD |
| CD127 BV421 | HIL-27-M21 | mouse IgG1 | 562437 | BD |
| PD-1 BV786 | EH12.1 | mouse IgG1 | 563789 | BD |
| HLA-DR BV786 | L243 | mouse IgG2a | 307642 | Biolegend |
| TIM-3 BV421 | F38-2E2 | mouse IgG1 | 345008 | Biolegend |
| LAG-3 PE-Cy7 | 3DS223H | mouse IgG1 | 25-2239-42 | eBioscience |
| FoxP3 PE | PCH101 | rat IgG2a | 12-4776-42 | eBioscience |
| Ki67 FITC | B56 | mouse IgG1 | 556026 | BD |
| CTLA-4 PE-CF594 | BNI3 | mouse IgG2a | 562742 | BD |
| TNF-α FITC | Mab11 | mouse IgG1 | 554512 | BD |
| IL-2 PE-Cy7 | MQ1-17H12 | rat IgG2a | 25-7029-42 | eBioscience |
| IFNγ APC | B27 | mouse IgG1 | 554702 | BD |
| Granzyme B PE-CF594 | GB11 | mouse IgG1 | 562462 | BD |
| PD-1 (unconjugated) | EH33 | mouse-IgG2a | 43248S | Cell Signaling |
| CD8 (unconjugated) | C8/144B | mouse IgG1 | M7103 | DAKO |
| CD3 (unconjugated) | polyclonal | rabbit | A0452 | DAKO |
| FoxP3 (unconjugated) | 236A/E7 | mouse IgG1 | ab20034 | Abcam |
| Granzyme B (unconjugated) | GRB-7 | Mouse IgG2a | M7235 | DAKO |

**Supplementary Table 2. Antibody specifications**
