# Supplementary material for: Efficacy of PD-1 blockade in cervical cancer is related to a CD8+FoxP3+CD25+ T-cell subset with operational effector functions despite high immune checkpoint levels
Source: J Immunother Cancer. 2019 Feb 12;7:43. doi: 10.1186/s40425-019-0526-z (PMC6373123; doi:10.1186/s40425-019-0526-z)
Supplement: Supplementary file 3 — Figure S1. Gating strategies. (PDF 159 kb) [file 40425_2019_526_MOESM3_ESM.pdf]

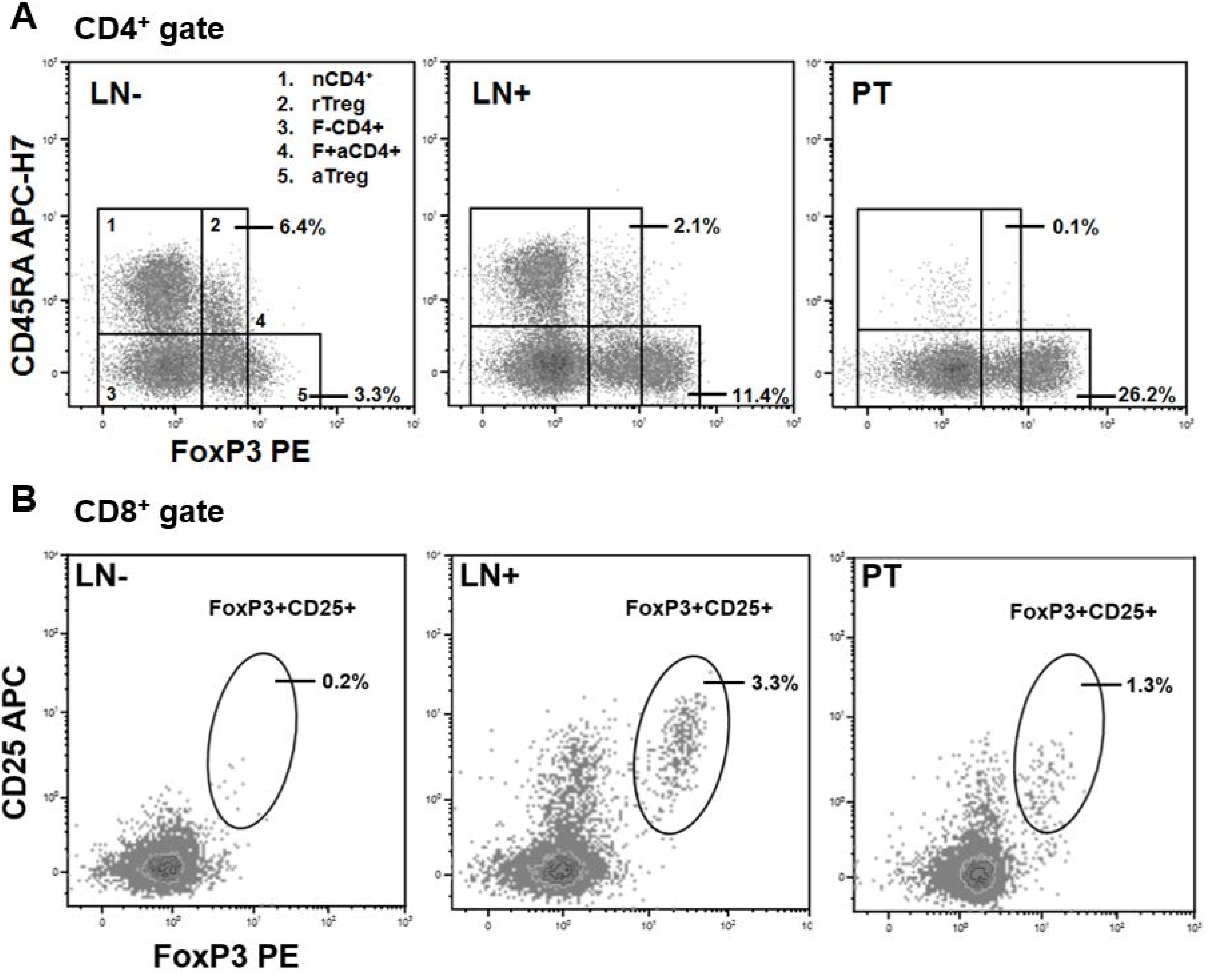
A
CD4+ gate
LN-
1. nCD4+
2. rTreg
3. F-CD4+
4. F+aCD4+
5. aTreg
6.4%
3.3%
LN+
2.1%
11.4%
PT
0.1%
26.2%
CD45RA APC-H7
FoxP3 PE
B
CD8+ gate
LN-
FoxP3+CD25+
0.2%
LN+
FoxP3+CD25+
3.3%
PT
FoxP3+CD25+
1.3%
CD25 APC
FoxP3 PE

Supplementary Figure 1. Gating strategies. (A) Gating strategy for different $CD4^+$ T cell subsets in representative examples of LN- (from patient 10), LN+ (from patient 10) and PT (from patient 7) as described by Miyara *et al*. (30). **(B)** Gating strategy for $CD8^+FoxP3^+CD25^+$ T cells is provided in representative examples of LN- (from patient 10), LN+ (from patient 10) and PT (from patient 7).
