# Supplementary material for: Efficacy of PD-1 blockade in cervical cancer is related to a CD8+FoxP3+CD25+ T-cell subset with operational effector functions despite high immune checkpoint levels
Source: J Immunother Cancer. 2019 Feb 12;7:43. doi: 10.1186/s40425-019-0526-z (PMC6373123; doi:10.1186/s40425-019-0526-z)
Supplement: Supplementary file 4 — Figure S2. Co-expression of immune checkpoint receptors on CD8+ T-cell subsets. (PDF 1990 kb) [file 40425_2019_526_MOESM4_ESM.pdf]

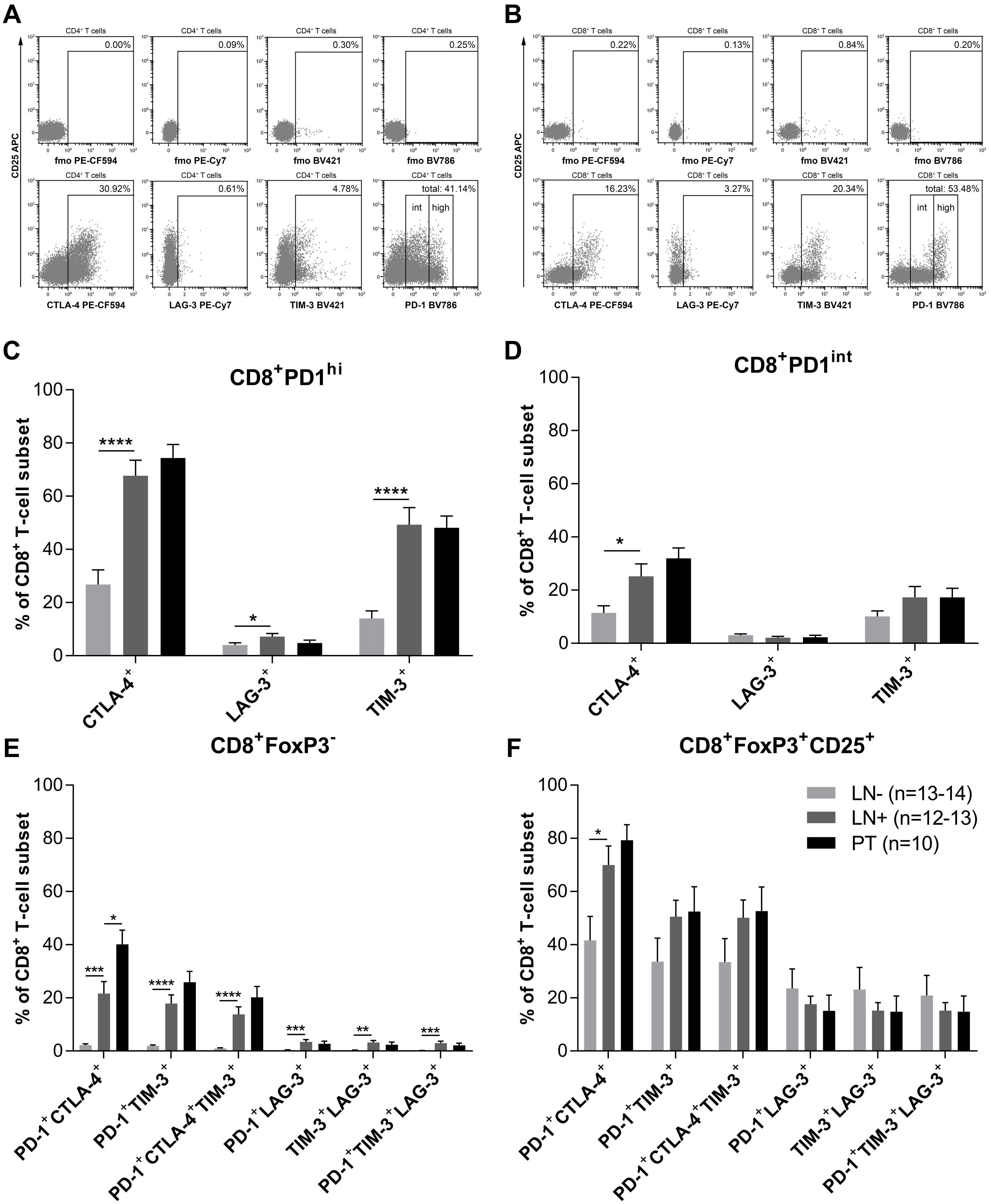
A
B
C
D
E
F
CD4+ T cells
CD8+ T cells
CD25 APC
0.00%
0.09%
0.30%
0.25%
30.92%
0.61%
4.78%
total: 41.14%
0.22%
0.13%
0.84%
0.20%
16.23%
3.27%
20.34%
total: 53.48%
int
high
fmo PE-CF594
fmo PE-Cy7
fmo BV421
fmo BV786
CTLA-4 PE-CF594
LAG-3 PE-Cy7
TIM-3 BV421
PD-1 BV786
CD8+PD1hi
CD8+PD1int
CD8+FoxP3-
CD8+FoxP3+CD25+
% of CD8+ T-cell subset
CTLA-4+
LAG-3+
TIM-3+
PD-1+CTLA-4+
PD-1+TIM-3+
PD-1+CTLA-4+TIM-3+
PD-1+LAG-3+
TIM-3+LAG-3+
PD-1+TIM-3+LAG-3+
LN- (n=13-14)
LN+ (n=12-13)
PT (n=10)

Supplementary Figure 2. Co-expression of immune checkpoint receptors on CD8$^+$ T-cell subsets. Gating strategy is provided in a representative LN+ for immune checkpoints CTLA-4, LAG-3, TIM-3, and PD-1 on **(A)** non-Tregs CD4$^+$ T cells and **(B)** CD8$^+$ T cells based on fluorescence minus one (FMO), both plotted against CD25. Co-expression of immune checkpoints on **(C)** PD-1^{hi} CD8$^+$ T cells and **(D)** PD-1^{int} CD8$^+$ T cells. **(E)** Elevated levels of multiple immune checkpoints on CD8$^+$FoxP3$^-$ T cells in LN+ *vs.* LN-. **(F)** High frequencies of CD8$^+$FoxP3$^+$CD25$^+$ T cells co-express immune checkpoints in LN-, LN+ and PT. Error bars represent standard error of the mean. LN-: *n*=13-14, LN+: *n*=12-13, PT: *n*=10. *P=0.01 to 0.05, ***P*=0.001 to 0.01, ****P*=0.001 to 0.0001, *****P*<0.0001.
