# Supplementary material for: Efficacy of PD-1 blockade in cervical cancer is related to a CD8+FoxP3+CD25+ T-cell subset with operational effector functions despite high immune checkpoint levels
Source: J Immunother Cancer. 2019 Feb 12;7:43. doi: 10.1186/s40425-019-0526-z (PMC6373123; doi:10.1186/s40425-019-0526-z)
Supplement: Supplementary file 5 — Figure S3. The IFNγ ELISPOT reactivity in LN-, LN+ and PT single cell suspensions. (PDF 380 kb) [file 40425_2019_526_MOESM5_ESM.pdf]

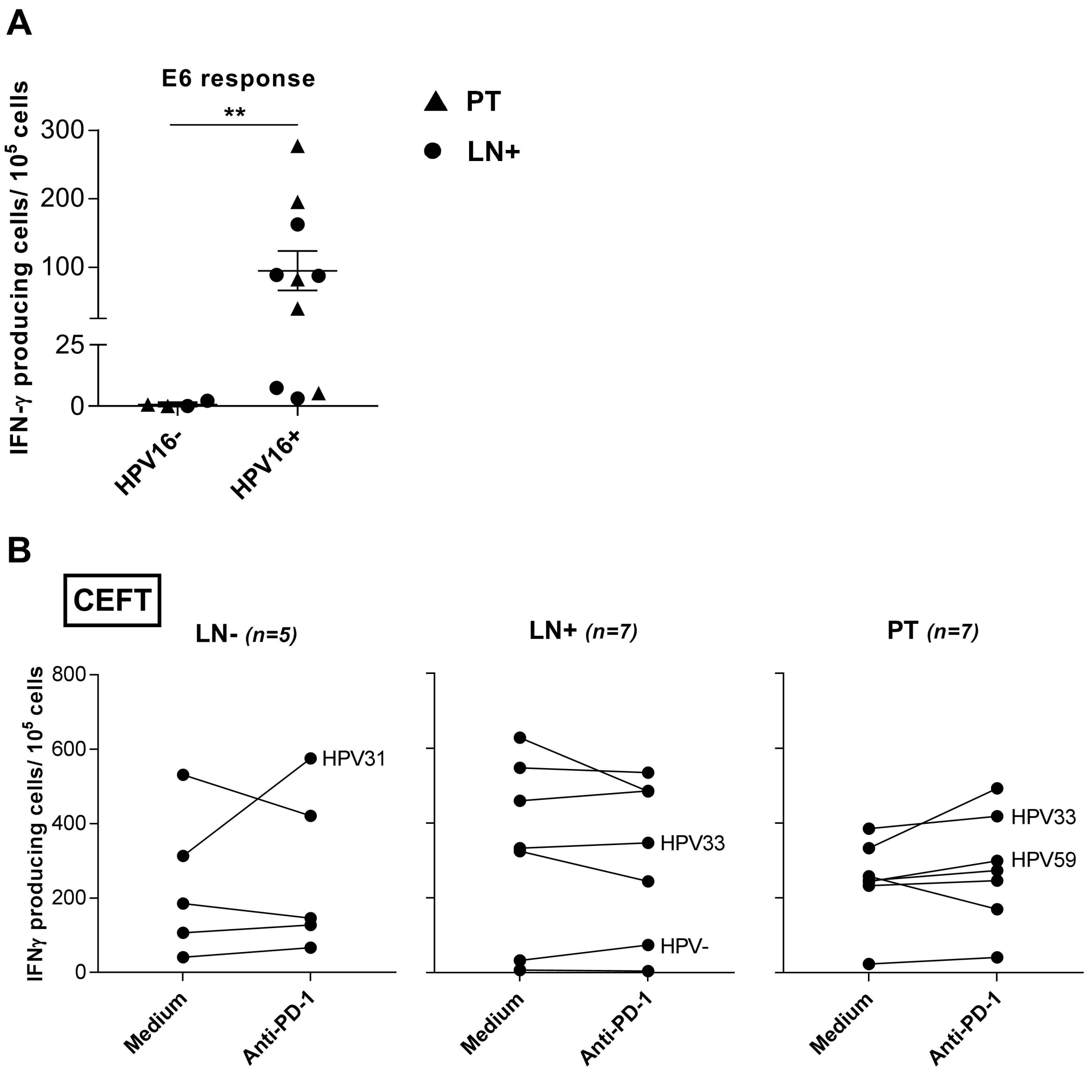
A
E6 response
**
PT
LN+
IFN-γ producing cells/ 10^5 cells
300
200
100
25
0
HPV16-
HPV16+
B
CEFT
LN- (n=5)
LN+ (n=7)
PT (n=7)
IFNγ producing cells/ 10^5 cells
800
600
400
200
0
HPV31
HPV33
HPV-
HPV33
HPV59
Medium
Anti-PD-1
Medium
Anti-PD-1
Medium
Anti-PD-1

Supplementary Figure 3. The IFNγ ELISPOT reactivity in LN-, LN+ and PT single cell suspensions. (A) IFNγ T cell reactivity against HPV16 E6 was only observed in HPV16+ patients (*n*=5 for LN+; *n*=5 for PT) rather than in patients with other HPV infections (*n*=2 for LN=; *n*=2 for PT). **(B)** The IFNγ ELISPOT reactivity against positive recall control CEFT is expressed as number of spots per 100.000 T cells. No consistent effect of PD-1 blockade is detected in LN- (*n=5)*, LN+ (*n*=7) or PT (*n*=7). HPV-types other than 16 are labeled in the graphs.
