# Supplementary material for: Efficacy of PD-1 blockade in cervical cancer is related to a CD8+FoxP3+CD25+ T-cell subset with operational effector functions despite high immune checkpoint levels
Source: J Immunother Cancer. 2019 Feb 12;7:43. doi: 10.1186/s40425-019-0526-z (PMC6373123; doi:10.1186/s40425-019-0526-z)
Supplement: Supplementary file 6 — Figure S4. Cytokine expression upon anti-CD3 stimulation: CD8+ vs. CD4+ T cells. (PDF 822 kb) [file 40425_2019_526_MOESM6_ESM.pdf]

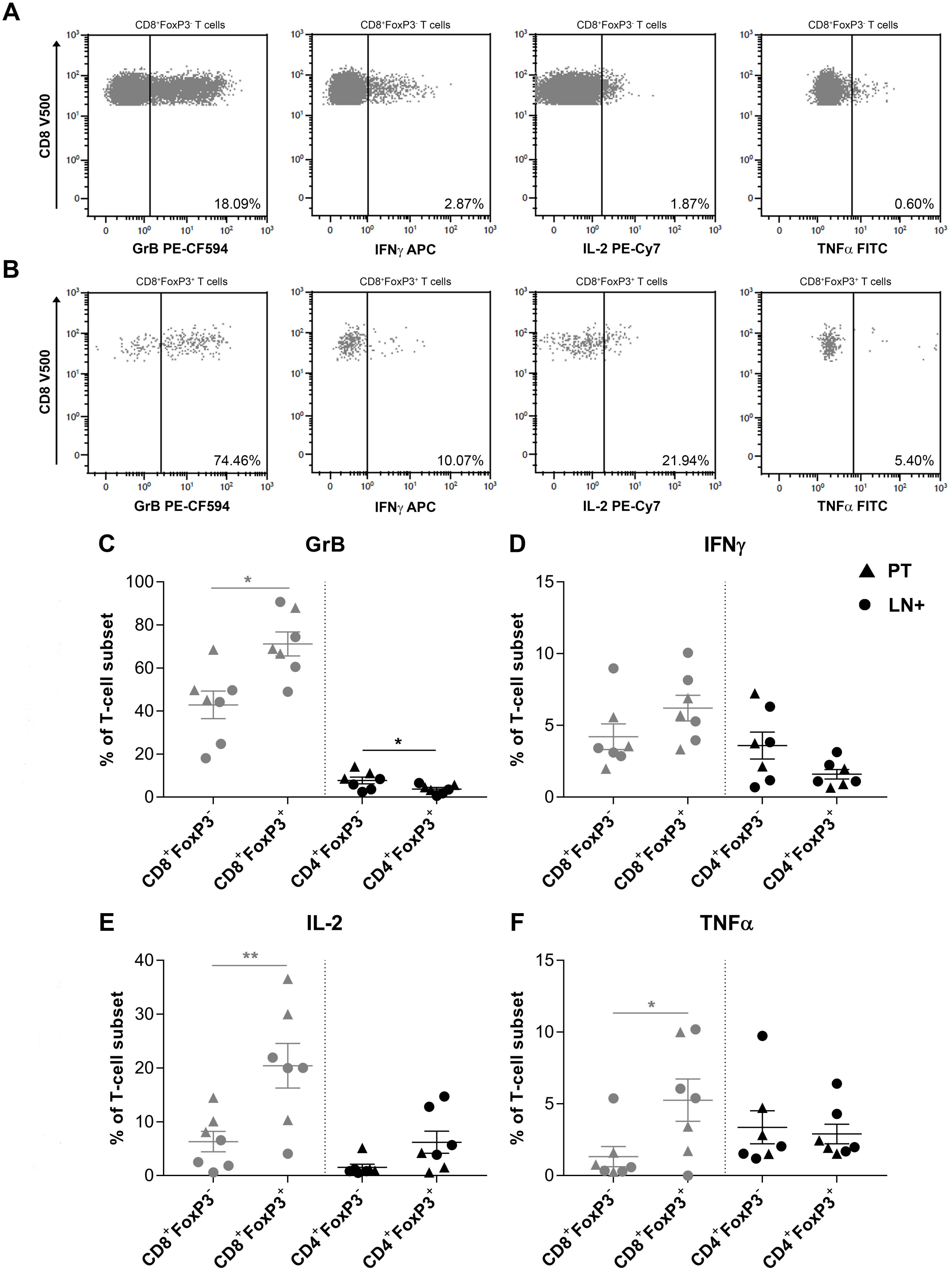

A
B
C
D
E
F
CD8+FoxP3- T cells
CD8+FoxP3+ T cells
CD8 V500
GrB PE-CF594
IFNγ APC
IL-2 PE-Cy7
TNFα FITC
18.09%
2.87%
1.87%
0.60%
74.46%
10.07%
21.94%
5.40%
GrB
IFNγ
IL-2
TNFα
PT
LN+
% of T-cell subset
CD8+FoxP3-
CD8+FoxP3+
CD4+FoxP3-
CD4+FoxP3+
*
**

Supplementary Figure 4. Cytokine expression upon anti-CD3 stimulation: CD8$^+$ *vs.* CD4$^+$ T cells. Dot plots showing representative gates for intracellular cytokines GrB, IFNγ, IL-2, and TNFα in **(A)** CD8$^+$FoxP3$^-$ T cells and in **(B)** CD8$^+$FoxP3$^+$ T cells present in LN+. Frequency of CD8$^+$ (grey) *vs.* CD4$^+$ (black, including Treg) T cells expressing intracellular **(C)** GrB, **(D)** IFNγ, **(E)** IL-2, and **(F)** TNFα upon o/n anti-CD3 stimulation in LN+ (n=4) and PT (n=3) samples. Error bars represent standard error of the mean. *P=0.01 to 0.05, **P=0.001 to 0.01.
